# Losses Disguised as Wins Affect Game Selection on Multiline Slots

**DOI:** 10.1007/s10899-018-9773-z

**Published:** 2018-05-05

**Authors:** Candice Graydon, Madison Stange, Mike J. Dixon

**Affiliations:** 1 Department of Psychology, University of Waterloo, Waterloo, ON Canada; 2 Gambling Research Lab, University of Waterloo, Waterloo, ON Canada

**Keywords:** Slot machines, Game preferences, Decision making, Losses disguised as wins, Payback percentage, Reinforcement rate

## Abstract

Multiline slots are exciting games that contain features which make them alluring. One such feature is a loss disguised as a win (LDW); wherein, players win less than they wager (e.g., bet 2 dollars, win back 50 cents), but this net loss is *disguised* by flashing graphics and winning sounds. Research to date concludes that LDWs are both rewarding and reinforcing. Here, we investigated whether LDWs affect players’ game selection. Thirty-two undergraduate students with experience playing slot machines played 100 spins on four games—two had positive payback percentages (115%) and two had negative payback percentages (85%) after 100 spins. For each payback percentage condition, there was a game with no LDWs and a game with a moderate number of LDWs. For the 100 spins, players could choose to play whichever game they wished. They then rated their preference for each game following the 100-spins and chose a game to continue playing. The majority of players preferred playing the positive payback percentage game with LDWs and chose to continue playing this game over the three other games. We conclude that in addition to LDWs being reinforcing and rewarding, LDWs do in fact influence game selection. We conclude that responsible gambling initiatives should educate players about LDWs.

## Introduction

Multiline slot machines are exciting games that are available in several nations worldwide. For a subset of gamblers, these games pose serious problems. By investigating the characteristics of these games, we can better understand the allure for these players. One such characteristic involves losses disguised as wins, or LDWs (Dixon et al. 2010). LDWs are outcomes where one wins back less than they wager (e.g., bet two dollars, win back fifty cents). Despite the net loss to the player, LDWs are accompanied by salient visual graphics and high fidelity winning sounds. A concern for gambling researchers is that if players misconstrue LDWs as wins, then they may drastically increase the apparent reinforcement rate of these games.

LDWs occur only on multiline games (e.g., Harrigan et al. 2011). Players tend to wager on the maximum number of playable lines (Templeton et al. 2015; Livingstone et al. 2008), and when doing so, they encounter more LDWs than actual wins. If LDWs are miscategorized as wins, players may believe that they won more often than they did in reality. Numerous studies have shown that at least some players do miscategorize LDWs as wins. Dixon et al. (2010) showed that novices somatically respond to LDWs as wins—their skin conductance responses (SCRs) following LDWs and wins were equivalent, both being higher than their SCRs following losses.

Dixon et al. (2014a) also showed that players behaviorally miscategorize LDWs as wins. Post-reinforcement pauses (PRPs) measure the time between the outcome delivery on one spin and the initiation of the next spin and serve as a behavioural indicator of how rewarding various outcomes are. Dixon and colleagues compared 2-credit payouts in a 1-line game (where this outcome was an actual win) versus a 20-line game (where a 2-credit payout was a net loss of 18 credits). In both games, participants’ PRPs were significantly longer for these 2-credit returns than for full losses (gains of zero credits) indicating they were rewarding. Crucially, participants’ PRPs following the 2-credit returns (the wins in single line game, the LDWs in 20-line game) were equally long (i.e., equally rewarding). Converging evidence that players behaviourally react to small wins and LDWs equivalently can be gleaned by measuring how hard players press the spin button. After losses, minimal force is applied. After large wins, more force is applied (presumably due to the excitement of the win). As with the PRPs, equivalent forces were applied following small wins and LDWs indicating that this type of loss was as exciting as a small win (Dixon et al. 2018). Finally, research has shown that LDWs lead players to verbally (cognitively) mislabel LDWs as wins/gains (Jensen 2011; Jensen et al. 2013; Graydon et al. Submitted), which leads them to overestimate how often they thought they won during a playing session (Jensen 2011; Jensen et al. 2013; Dixon et al. 2014a, b, 2015; Templeton et al. 2015). This latter result has been referred to as the LDW-triggered win-overestimation effect, which has been replicated with both novice and experienced gamblers.

The previous studies highlight that LDWs are rewarding. Recent research (Graydon et al. Submitted) has shown that LDWs can actually extend play. Following a 100-spin session participants were instructed that they could continue to play for as long as they wished or quit at any time. Unbeknownst to players, all subsequent outcomes were net losses—in one condition there was a moderate number of LDWs interspersed in the losing streak, in the other there were only a few. Gamblers persisted significantly longer if they experienced a moderate number of LDWs during the streak. Converging evidence for these findings comes from Leino et al. (2016) who examined player data from electronic gaming machines. They showed that players were more likely to continue playing following an LDW than following a regular loss. Thus, LDWs can lead players to continue gambling despite financial loss, and increase play durations.

An important question that arises then is, will players seek out and preferentially *choose* to play games with LDWs? In this experiment, participants were able to play four different games which crossed the presence/absence of LDWs with two different payback percentages (85 and 115%). Thus there were two “losing games” (one with and one without LDWs) where players would lose money and there were two “winning games” (one with and one without LDWs) where players would end up with a net profit. The four games appeared in the four quadrants of a single display. Players were allowed to play for 100 spins, and they could distribute these spins across the four games however they wished. After the 100 spins, players were given the choice to play whichever (of the four) games they wanted for a minimum of 10 additional spins and then could play for as long as they wished or quit at any time. (Unbeknownst to players, all subsequent spins on whatever game they chose were losses). Participants also rated how much they preferred playing each of the four games. We predicted that participants would prefer the “winning” games (115% payback) over “losing” games (85% payback). Crucially, however, we also predicted that players would prefer to play the games with LDWs over the games without LDWs. Overall we predicted that LDWs would work in conjunction with high payback percentages and lure players into preferentially choosing (and preferring) the 115% payback percentage game with LDWs. Further, even for those who happened to choose a low payback percentage game, we predicted that these players would choose to play an LDW (losing) game over a no LDW (losing) game.

Although our central predictions involved LDWs’ effects on player’s game choices and preferences, we also conducted exploratory analyses to see if they might persist for longer if they chose to play a game with LDWs than if they chose to play a game without LDWs.

## Method

### Participants

#### Recruitment/Selection

Thirty-six undergraduate students were recruited from the Department of Psychology’s Research Experience Group. Data from 3 participants was discarded prior to analyses due to equipment malfunctions and/or missing data, leaving a final sample of 33 participants. Prescreening ensured that all participants were: (1) 19 years of age or older; (2) not in treatment for problem gambling; (3) not in treatment for an anxiety disorder and/or taking medication for an anxiety disorder [a criteria necessary to maximize skin conductance levels which were not analyzed here]; and (4) had played a slot machine at least once in the past 12 months. Participants were tested in a single session where they were given the option to receive 10 dollars, 5 dollars plus half a course credit, or one course credit for their time. They were also given 20 dollars to play the slot machine and were informed that they could keep the cash remaining on the machine (end balance) up to a maximum of $40 once the playing session was over. In actuality, the most they could receive is $24. All study procedures/methods were reviewed and approved by the University’s Office of Research Ethics.

#### Canadian Problem Gambling Index

Prior to gameplay the Canadian Problem Gambling Index (Ferris and Wynne 2001) was administered using Quatrics (an online survey platform) to assess participants’ slots play frequency over the past year, problem gambling severity levels (via the Problem Gambling Severity Index), age, and gender. Data was missing for one participant. Using the interpretive cut-offs proposed by Currie, Hodgins, and Casey (2013), 16 participants were deemed non-problem gamblers (PGSI = 0) and 16 low-risk gamblers (PGSI 1–4). Ages ranged between 19 and 29 (*M* = 21.16, *SD* = 1.82) and included 24 (75%) females.

### Apparatus

#### Slot Machine Simulator

**Fig. 1:**
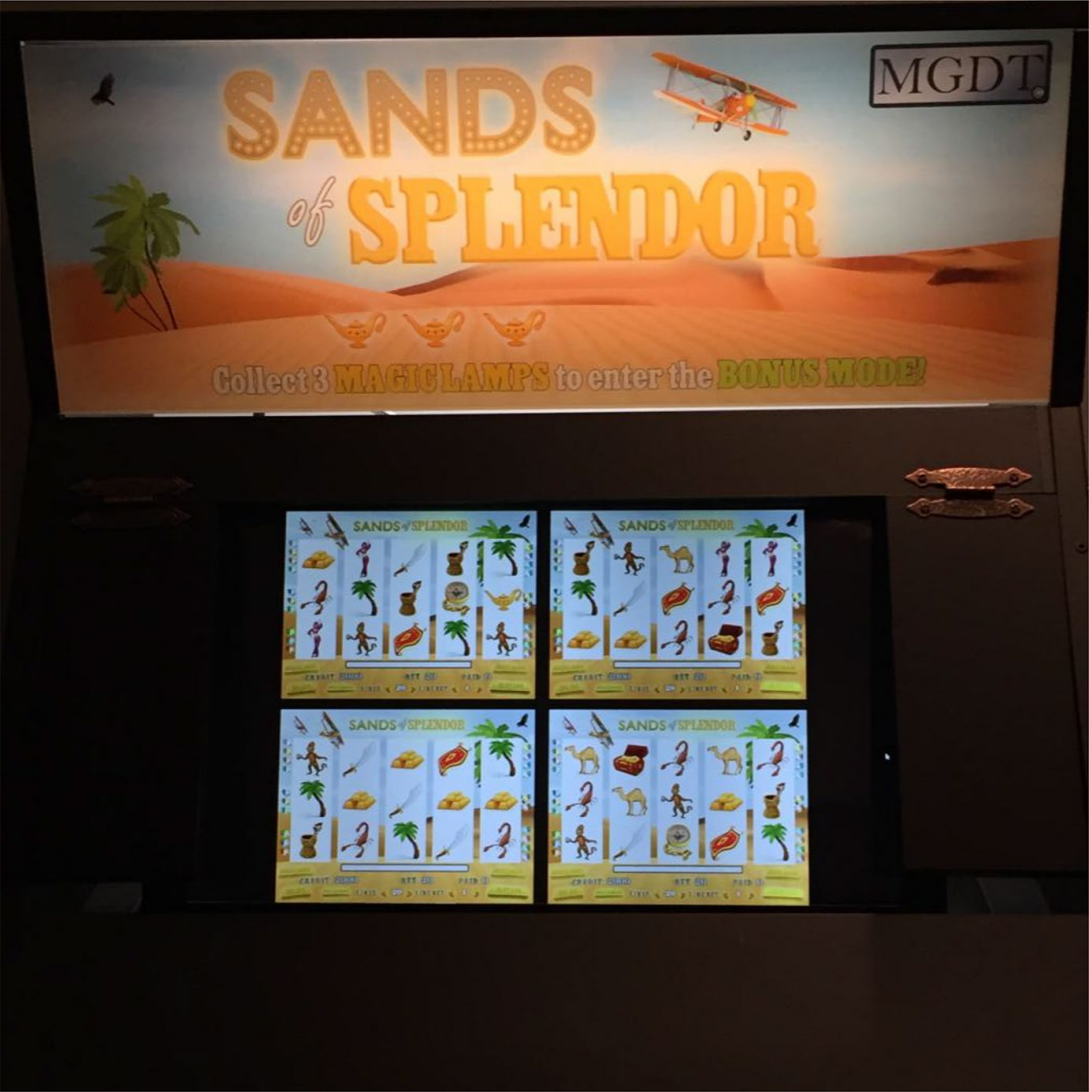
Picture of the four game simulator and slot machine cabinet

Figure 1 shows the slot machine, Sands of Splendor (SoS), used in this study. SoS is a desert-themed multi-line game (with 20 playable lines). The games were displayed on a Dell (Inspiron ONE2330) touch screen computer. In the first part of the playing session, four SoS games were displayed on the screen in the upper left and right, and lower left and right quadrants of the screen at any given time (see Fig. 1). In the second part of the playing session, one SoS game was displayed full-screen on the monitor. Participants interacted with the game by touching the spin buttons(s) on the screen using their dominant hand. For all games, participants wagered 1 credit per line on 20 paylines, for a total wager of 20 credits per spin. Participants could not change their wager during the study.

#### Slot Machine Cabinet

To maximize ecological validity, we built a custom slot machine cabinet (see Fig. 1). This cabinet was designed to make only the touch-screen portion of the monitor visible to participants. We placed custom-made glass on top of the cabinet. The back-lit graphics of the glass were patterned after SoS’s desert theme.

#### Slots Game Designs

Sounds in the game ranged from 0.3 to 13.48 s, and were patterned after commercially available slots games. In part one of the playing session, we used a two by two design for the four games, with payback percentage (85% PB, 115% PB) and LDW frequency (no LDWs, moderate LDWs) as the factors. We will refer to these four games as 85% PB no LDWs, 85% PB with LDWs, 115% PB no LDWs, and 115% PB with LDWs from hereon in. Each game had 19 real wins. In the no LDW games, there were zero LDWs. In the two LDW games, there were 14 LDWs (an LDW percentage mimicking a commercially available slot machine). The 85% payback percentage was chosen because it is the lowest payback percentage allowable in Ontario. The 115% positive expected value condition was chosen because it is a winning payback percentage that frequent players would (theoretically) have experienced at least on some occasions (i.e., when players are “up” at the end of a session they are more likely to be up by 115% than higher values associated with jackpots). Participants started with 2000 credits on each game ($20). The two 85% PB games would have ended with 1700 ($17) credits had participants played 100 spins exclusively on these games. The two 115% PB games would have ended with 2300 credits ($23) after 100 spins.

**Fig. 2:**
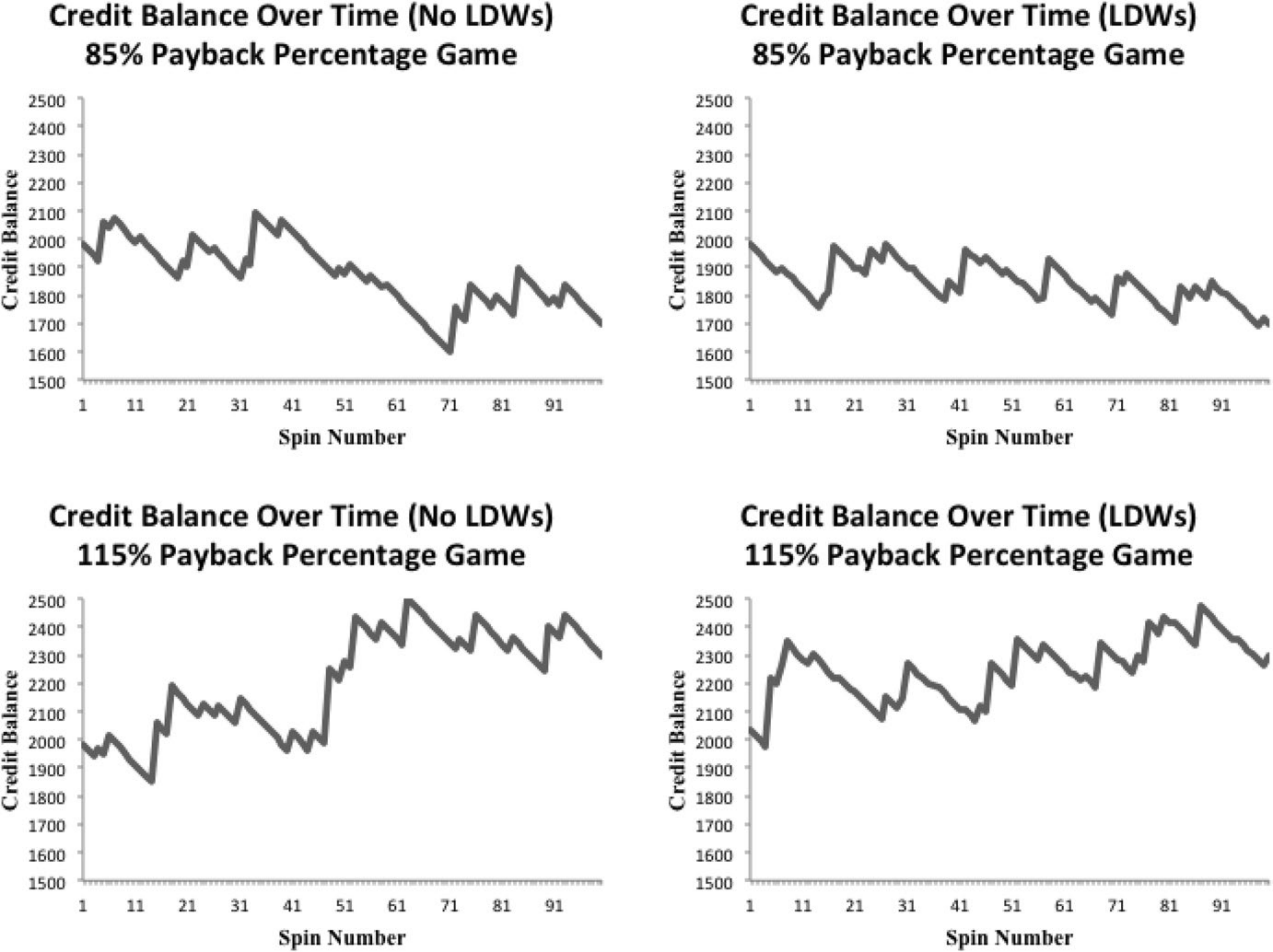
Credit balances for the four different games (85% PB no LDWs, 85% PB LDWs, 115% PB no LDWs, 115% PB LDWs) during the first part of the playing session

Figure 2 shows the credit balances (and win/LDW sizes) over time for the 4 different games. The median credit size and sound length for wins/LDWs in the 85% PB no LDW game were 74 credits and 6.46 s, respectively. The median credit size and sound length for wins/LDWs in the 85% PB with LDW condition were 33 credits and 2.95 s, respectively. The median credit size and sound length for wins/LDWs in the 115% PB no LDW condition were 84 credits and 7.32 s, respectively. Finally, the median credit size and sound length for wins/LDWs in the 115% PB with LDW condition were 52 credits and 4.58, respectively. Since LDWs lead to net losses (players get back less than their 20 cents per spin wager) we had to include larger wins in the no LDW conditions to maintain the payback percentages at fixed rates of 85 and 115%.

It is possible for the four games to be displayed in one of 24 possible orders of locations on the screen. We randomly sampled four of these possible orders, and randomly assigned participants to play one of these four orders. The orders were as follows (in order from upper left, upper right, lower left, and lower right of screen): (1) 85% PB no LDWs, 115% PB with LDWs, 115% PB no LDWs, 85% PB with LDWs, (2) 115% PB with LDWs, 85% PB no LDWs, 85% PB with LDWs, 115% PB no LDWs, (3) 85% PB with LDWs, 115% PB no LDWs, 85% PB no LDWs, 115% PB with LDWs, (4) 115% PB no LDWs, 85% PB with LDWs, 115% PB with LDWs, 85% PB no LDWs.

Prior to this playing session, participants played five practice spins on each game to familiarize them with the games. In the two no LDW games, there was one win in the five spins. In the two LDW games, there was one win and one LDW. After the 100-spin playing session, participants had to choose to play one of the four games (extinction phase where persistence was measured; see procedure). They had to play a minimum of 10 spins, after which they could play for as long as they wished or quit at any time. In the 10 spins there were two moderate sized wins (88–96 credits) in the 85% PB percentage games. There was one moderate win (96–99 credits), and one larger win (118–124 credits) in the 115% PB percentage games. The two LDW games also had one LDW (9–12 credits) in the 10 spins. These spins were included for two reasons: (1) to familiarize participants with playing one game on the screen, and (2) to make it appear as if they were playing the same game that they chose during the 100-spin session. All outcomes following these 10 spins, however, were losses.

#### Skin Conductance Responses (SCRs)

Skin conductance was recorded, but recordings were compromised by movement artifacts associated playing the four games.

### Materials

#### Slot Machine Tutorial

Participants viewed a short (7 min and 16 s) visual tutorial (with narration) that showed all of the features of SoS (including the “bet” and “paid” counters which if attended to, indicate the true nature of LDWs).

#### Measures^1^

Scales were administered on Qualtrics. For each game participants were asked “How would you rate your preference for the highlighted game (below) on a scale from 0 (I did not enjoy this game at all) to 100 (I enjoyed this game the most).” A yellow box highlighted the game of interest. The participant answered this game preference question four times, once per game. Participants responded to the question by sliding a bar on a 100 mm visual analogue scale.

### Procedure

Participants came to a waiting area adjacent to the testing room. After signing an informed consent form, participants washed their hands to maximize the quality of the SCL recording (not analyzed in this study).

Participants completed pre-game scales that were collected for reasons peripheral to this experiment (see footnote 1) then watched the slots tutorial. Participants then sat at the slot machine, and we attached the skin conductance electrodes to the index and ring fingers of their non-dominant hand.

We reminded participants of the key features of the games by pointing to the relevant information on the screen (e.g., bet and paid counters). They were first told that there were four games on the machine, and that they would be wagering 20 cents per spin on any game that they played. They were informed that they would be playing 100 spins in total and would be asked some questions before and after the playing session. They were also informed that they did not need to count the spins; rather, a researcher would let them know when there were two spins remaining. They were instructed that they could not change their wager or the number of lines played during the game; that each game was preset to a balance of 2000 credits or $20 per game; and that they could keep the remaining balance on whichever game they chose to play at the end of the playing session (if any) up to a maximum of $40.

Participants played 5 practice spins on each game, (in clockwise fashion starting with the game in the upper right). Participants were then informed that they would play 100 spins, and could play whatever game they would like on each spin. Participants played 98 spins on whichever game they wanted per spin, and then the researcher informed them when they had two spins left. At 100 spins, the researcher asked participants “which game would you like to keep playing”. After answering items peripheral to this study they then rated how much they enjoyed playing each of the four games.

While participants completed the aforementioned questions, the researcher loaded the participant’s “preferred” simulator game on to the slot machine. The researcher set the starting balance on the participant’s new game to the end balance (rounded up to the nearest 100 credits) of the game they chose to play in the previous 100-spin session (i.e., if they chose to play the 85% PB no LDW game, and the end balance on this game was 1623 credits, then the start balance of the new game was set to 1700 credits).

Participants played 10 spins on their preferred game whereupon the researcher handed participants a chit that stated, “at this point during the playing session, you can continue to play for as long as you want. You can choose to stop playing at any time.” The chit also stated that once they were finished, to gently remove the SCL electrodes. The researcher recorded the number of persistence spins. After quitting, players filled out more peripheral items (see footnote 1). Participants signed a receipt for any cash obtained from the end balance on the machine and were given their course credit and/or cash for participating in the study. They were debriefed, and given two responsible gambling brochures, a wallet card and a pencil with the problem gambling helpline’s number on it, and information for a local community crisis/mental health/addiction hotline.

## Results

### General Analytical Notes

Given the small sample size and relatively limited range of players with any gambling problems for this experiment, we did not include PGSI level as a factor in our analyses.

### Game Choice

**Fig. 3:**
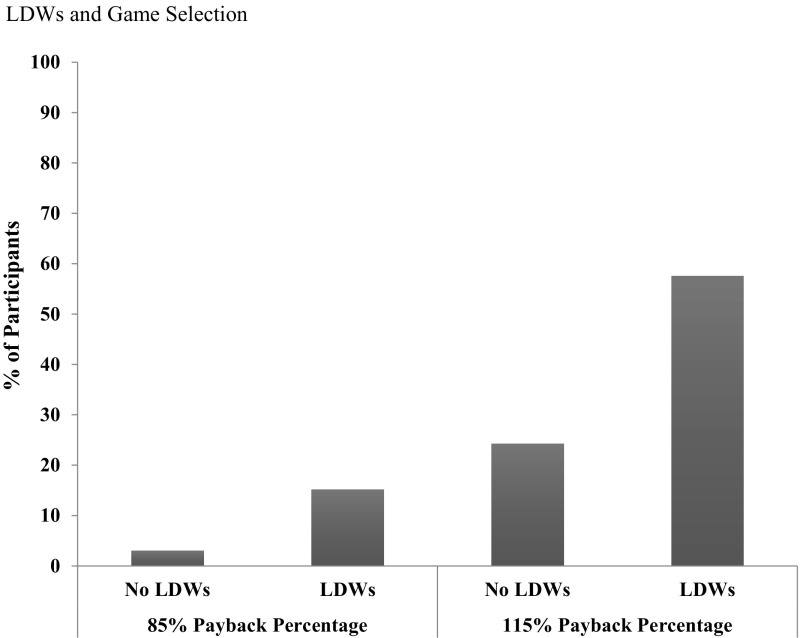
Percentages of participants who chose to play each of the four games (85% PB no LDWs, 85% PB moderate LDWs, 115% no LDWs, 115% moderate LDWs) following the 100 spin playing session

Three percent chose to play the 85% PB no LDW game, 15% chose to play the 85% PB LDW game, 24% chose to play the 115% PB no LDW game, and 58% chose to play the 115% PB LDW game. These percentages, depicted in Fig. 3, clearly show that the majority of individuals chose to play the 115% PB with LDWs game. To statistically verify these preference patterns we first analyzed the frequency of participants’ game choices using a Chi square goodness-of-fit test (with the expected value set to 25% for each game choice). This test was significant, *Χ*^*2*^*(3)* = 21.67, *p* < .001 indicating that equal numbers of participants did not choose each of the four games.

Next, we ran pairwise comparisons using restricted Chi squared tests (with null hypothesis expected values of 50%, or chance). The key comparison was between the two most popular games (the 115% with LDWs and the 115% without LDWs). Significantly more participants chose to play the 115% LDW game over the 115% no LDW game *Χ*^*2*^*(1)* = 4.48, *p* = .034. (By extension, the 115% LDW game was also chosen significantly more often than the remaining two games, which were chosen by even fewer participants). Importantly, there was no significant difference between the proportions of participants who chose to play the 115% no LDW game and the 85% LDW game, *Χ*^*2*^*(1)* = .69, *p* = .41. Significantly more participants chose to play the 115% no LDW game over the 85% no LDW game, *Χ*^*2*^*(1)* = 5.44, *p* = .02. There was no significant difference in the proportions of participants who chose to play the 85% no LDW game and the 85% LDW game, *Χ*^*2*^*(1)* = 2.67, *p* = .10.

### Game Preference Ratings

**Fig. 4:**
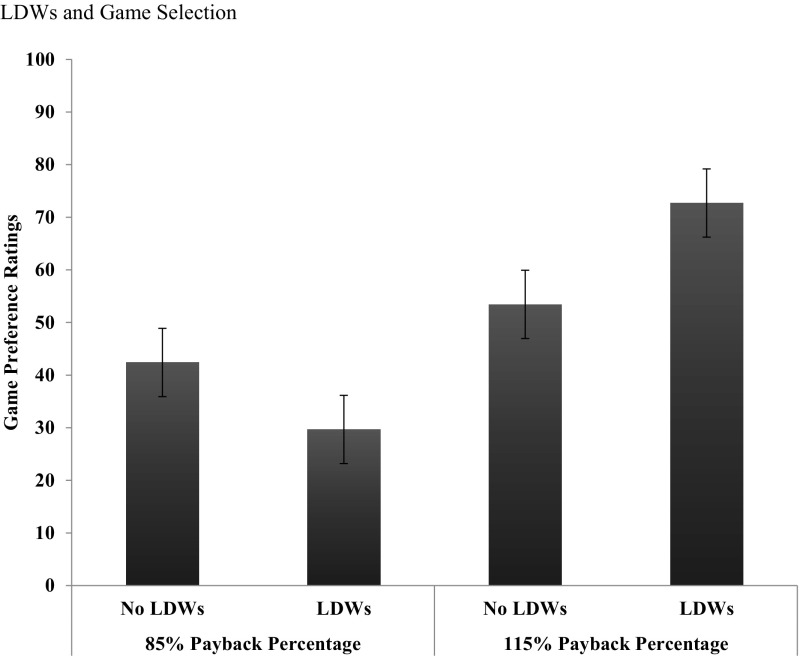
Participants’ mean preference ratings for each of the four games (85% PB no LDWs, 85% PB LDWs, 115% PB no LDWs, 115% PB LDWs). Error bars represent Masson and Loftus (2003) 95% confidence intervals

Figure 4 shows participants’ mean preference ratings for each of the games. One outlier (± 2 SD above/below the mean) was removed prior to analyses. Participants’ preference ratings for each game were analyzed with a two-way ANOVA with payback percentage (85, 115%) and LDW frequency (LDWs, no LDWs,) as the repeated measures factors. There was a significant main effect of payback percentage, *F*(1, 26) = 60.80, *p* < .001, *MSE* = 324.63, $\eta_{p}^{2}$ = .70. Overall, participants preferred playing the higher payback games (*M* = 63.07, *SE* = 3.01) to the lower payback percentages games (*M* = 36.04, *SE* = 3.11). There was no significant main effect of LDW frequency, *F*(1, 26) = 1.34, p = .26, *MSE* = 214.07, $\eta_{p}^{2}$ = .049, but importantly there was a significant payback percentage by LDW frequency interaction, *F*(1, 26) = 25.43, *p *< .001, *MSE* = 271.79, $\eta_{p}^{2}$ = .49.

To explore the interaction, we conducted paired-samples *t* tests evaluated against a Bonferroni correction of p/2 = .025. For each payback percentage condition (85, 115%), we compared the mean enjoyment ratings for the LDW vs no LDW games. For the 85% games, there was no significant difference (LDW game *M* = 30.39, *SD* = 19.66, no LDW game *M* = 40.89, *SD* = 20.14), *t*(27) = 2.34, *p* = .027, *SE*_*diff*_ = 4.49. For the 115% payback percentage games, participants enjoyed playing the LDW game (*M* = 74.59, *SD* = 19.80) more than the no LDW game, (*M* = 53.28, *SD* = 19.62), *t*(28) = 4.77, *p* < .001, *SE*_*dif*_ = 4.47.

### Persistence

Only one participant played the 85% PB no LDW game, preventing us from including this game in parametric analyses. The remaining participants’ persistence scores from their chosen game were first analyzed using a univariate ANOVA, with game chosen (85% PB with LDWs, 115% PB no LDWs, 115% PB with LDWs) games as the between subjects levels. While a trend was observed wherein persistence was highest for those who chose the 115% PB with LDWs game (n = 19, *M* = 17.00, *SD* = 12.35), followed by the 115% PB no LDW game (n = 8, *M* = 10.88, *SD* = 4.58), then the 85% with LDWs (n = 5, *M* = 7.40, *SD* = 5.08) game, the main effect of game choice was not significant, *F*(2, 29) = 2.26, *p* = .12, *MSE* = 103.24, $\eta_{p}^{2}$ = .14. Statistical power for this analysis was only .42.

## Discussion

As predicted, payback percentage and LDW frequency both had an effect on players’ game choices and game preferences. The majority of players chose to play the high payback percentage game with LDWs over all other games. Importantly, we found no significant difference between the number of players who chose to play the no LDW game with a high (winning) payback percentage and the low payback percentage game with LDWs. These results suggest that LDWs, which are monetary *losses*, do in fact affect players’ gambling behaviours (specifically, their game selections). We also found that gamblers preferred playing the games that had a positive expected value (115% payback percentage) over games that had a negative expected value (85% payback percentage). One limitation of our design, however, is that since participants could play as many spins as they wished on any game, they may not have played a sufficient number of spins on a given game to actually experience these positive or negative expected values. For instance, if one only played five spins on the no LDW 115% PB game, they would be “down” after those five spins. Despite this, as predicted, we did find that gamblers preferred playing the high payback percentage games over games the low payback percentage games. Importantly, however there was an interaction with whether the game had LDWs or not. When players played “losing” games, they did not report preferring the game with LDWs over the game with no LDWs. With the high PB “winning” games, however, they preferred playing the games with LDWs to the game with no LDWs. These results suggest that LDWs do affect players’ preferences when they are winning, or colloquially speaking, “up” in a game. Finally, while not significant, there was a trend showing that players persisted longer if they are “up” and are experiencing LDWs. In sum, the results of this experiment, (when combined with previously reviewed research), suggests that players somatically, behaviourally, and cognitively (via verbal labeling) miscategorize LDWs as wins rather than correctly categorizing them as losses; that players find LDWs rewarding and reinforcing, which affects their gambling behaviour despite financial loss; and that LDWs affect players’ game choices and preferences.

There are some limitations to this study. First, we only used a sample of relatively inexperienced slot machine gamblers. We did this as a first assay to see how payback percentage and LDWs could influence players’ game choices and preferences with little pre-existing experience or gambling problems. Future research should evaluate whether these results/effects would hold for a sample of experienced gamblers, and those with various levels of problem gambling symptomatology. Given that experienced gamblers show a LDW-triggered win-overestimation effect, and prefer playing the maximum number of playable lines on multiline games (which have more LDWs), we would predict that they would also choose to play games with more LDWs than games with fewer LDWs while they are up or “winning”.

Additional limitations surround the design of our slot machine game and various conditions. For example, in an attempt to make participant’s play sessions equitable, we set the bet size for participants throughout the study. It is possible that this format of play made the study somewhat artificial for some participants, as participants are free to choose and modify their wager in real-world slot machine games. Additionally, our experimental design necessitated systematic differences in outcome magnitude: in order to equate the overall balance for each of the games in each PB% condition, the no LDW conditions had larger median credit gain sizes than the LDW conditions. Perhaps this difference in credit gain size may explain the unexpected finding of participants’ greater preference for the no LDW game in the 85% PB condition. However, this comparison failed to reach statistical significance when Bonferroni corrected, and participants did not prefer the no LDW game over the LDW game in the 115% PB condition. Therefore, it seems that LDWs are a driving force behind participant game preferences in multiline slot machines. Future research should further explore the interaction between PB% and the number of LDWs experienced on participants’ gambling behaviour.

Recall, that we failed to show any statistically significant differences in players game preferences (between the no LDW and LDW games) when players were losing money. On most slot machine games, there is a certain level of volatility. There are times when a player is “up”, and other times when a player is “down”. While all slot machines are programmed such that a player loses in the long run, one may predict (given that there are a lot of erroneous cognitions surrounding slots games), that players may choose to continue playing a game if they are winning or “up” rather than a game where they are losing or “down”. In Ontario, payback percentages on games vary between 85 and 98%. We used the minimum payback percentage available, so players were losing 15%. Given that the average payback percentage is approximately 93% in Ontario (Personal Correspondence with Dr. Kevin Harrigan 2017), it would be interesting to see whether we would observe a shift in players’ preferences at this payback percentage—namely, whether they would start preferring the LDW over no LDW game if they were still losing, but not as much. It is possible that there was a “floor” effect, where players just did not prefer the losing games all together. Another limitation of this study is that, while players are “up” at times, no slots game, if played over an extended play period, actually has a positive payback percentage. Future research should evaluate whether the preference for the LDW over no LDW games while winning would still hold if at a higher than 85% payback percentage—say 98%, or the upper limit—where participants are still losing money. It is important to note, however, that slot machines’ payback percentages are calculated over a very large number of spins. These machines are quite volatile and frequent players can easily cite many instances when they left being “up” (as well as many more being “down”). Despite these concerns, overall we conclude that LDWs do have some effects on players’ game choices and preferences. Future research should explore whether LDWs affect players’ game preferences on the actual casino floor.

One implication of players choosing to play games with LDWs is that it could lead them to continue gambling despite financial loss. Thus, from a responsible gambling perspective, it is imperative to develop initiatives to inform players about LDWs early in one’s gambling career. This could be done by using educational animations (see Graydon et al. 2017); responsible gambling brochures; or demonstrations via kiosks at gambling venues. The later initiative has been employed by the Ontario Lottery and Gaming Corporation (OLG) PlaySmart program—where responsible gambling (RG) representatives have been touring select gambling venues to explain the randomness of slot machine games. A simple extension of this initiative would be to explain other structural characteristics as well, including LDWs. To conclude, future research should evaluate methods to translate knowledge about the potential (negative) consequences of LDWs to players.

## Footnotes

1 Several measures were taken that were peripheral to this study. Subjective mood and arousal (Bradley and Lang 2004) were taken prior to the 100 spin session, following the 100 spin session, and following the persistence spins. Desire to gamble (Young et al. 2008) and gambling urge (Raylu and Oei 2004b) were measured at the same 3 time-points. Prior to the gambling session, we administered the Depression, Anxiety, and Stress Scale (DASS 21; Lovibond and Lovibond 1995; Henry and Crawford 2005); Positive and Negative Affect Schedule (PANAS; Watson et al. 1988; Crawford and Henry 2004); Gambling Related Cognitions Scale (GRCS; Raylu and Oei 2004a); and Gambling Motivation Scale (GMS; Chantal et al. 1994). Following the gambling session, we administered the Game Experience Questionnaire (Poels et al. 2007) Rational-Experiential Inventory (REI; Pacini and Epstein 1999); Cognitive Reflection Test (CRT; Frederick 2005); Actively Open-Minded Thinking scale (AOT; Stanovich and West 1997); Adult ADHD self-report scale (ASRS; Kessler et al. 2005); and the Attention Related Cognitive Errors Scale (ARCES; Carriere et al. 2008).
